# MDCT Diagnosis of Symptomatic Incarceration of the Right Ileocecocolic Mesentery Within a Complete 360-Degree Whirl of the Great Omentum: Follow the Vessels!

**DOI:** 10.5334/jbr-btr.1182

**Published:** 2016-09-19

**Authors:** Bruno Coulier, Guillaume Verlynde, Frédéric Pierard

**Affiliations:** 1 Clinique Saint-Luc, Bouge, Belgium

**Keywords:** Greater omentum, abdomen, MDCT

## Case Report

**Figure 1:**
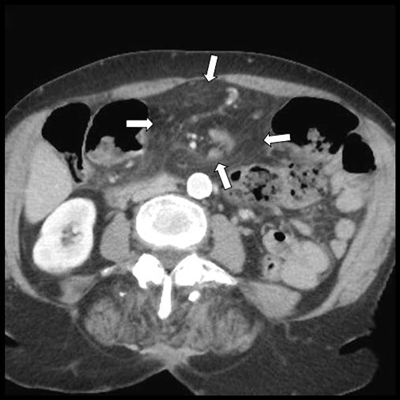


**Figure 2:**
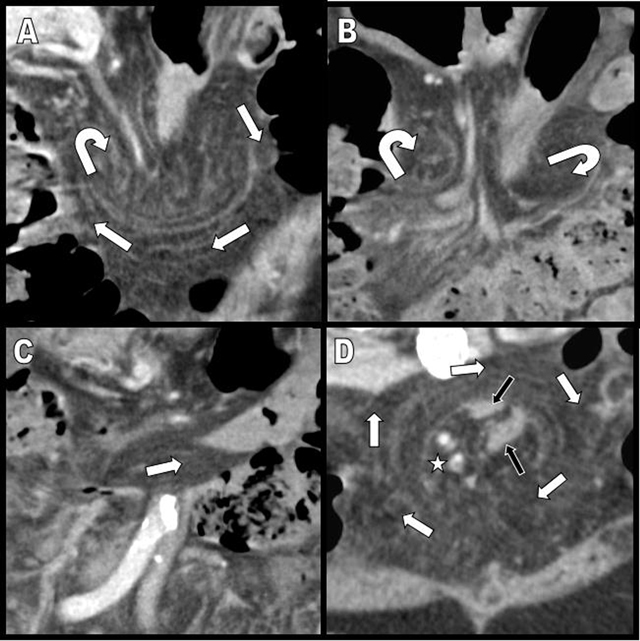


**Figure 3:**
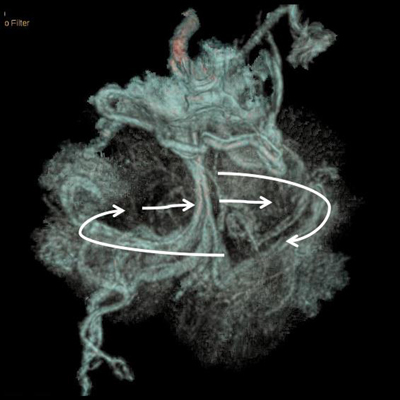


A 74-year-old female was referred to the emergency department with persistent abdominal pain. Five months earlier, she had experienced acute small bowel intestinal obstruction necessitating emergency laparotomy. This occlusion was caused by anterior parietal adherences related to recurrent previous abdominal surgery. Contrast-enhanced MDCT excluded now significant dilatation of the gut but demonstrated an unusual accumulation of abdominal fat incarcerating mesenteric vessels and bowel segments in the midline of the anterior mesogastrium (Figure 1, white arrows). Careful multiplanar analysis (Figure 2A, B, C shows the antero-posterior coronal MPR views, and Figure 2D shows the axial oblique MPR view) revealed the omental nature of the encircling fatty structure through the clear delineation of the encircling omental vessels (white arrows). This 360-degree whirl of the greater omentum was incarcerating the right ileocecocolic mesenteric vessels (white star); the two incarcerated and compressed bowel segments were identified as the terminal ileum and the transverse colon (black arrows). Selective volume-rendering reconstruction confirmed the whirl (Figure 3). Despite the fact that there was no critical dilatation nor suffering signs of the gut at the time of MDCT, the risk of occlusion was nevertheless considered important, and it seemed unlikely that this complete omental whirl would resolve spontaneously. This caution justified laparotomy.

## Comment

The greater omentum (GO) is a large free-hanging apron arising from the gastric greater curvature, crossing the transverse colon and descending in front of the hollow viscera. The GO has considerable mobility and may move freely all around the peritoneal cavity, and its average length is statistically significantly longer in females than in males, particularly its left portion.

Because of its mobility length and superficial position in the peritoneal cavity, the GO is also the most statistically exposed structure to protrude into hernias of the antero-superior abdominal wall and that from the diaphragm to the inguinal canal.

The MDCT identification of the normal GO, which is a nearly purely fatty structure, represents a challenge for the radiologist who ignores the diagnostic key. This key is the peculiar vertical vascular network of the GO which constitutes its unique landmark for current, prompt identification during MDCT.

The vascularization of the GO depends from the right and left gastroepiploic arteries which pass tortuously along the gastric greater curvature. Six to fourteen epiploic arteries originate at a right angle from these right and left gastroepiploic arteries and descend mostly at a right angle to the GO margin, where they bifurcate and may anatomose. The venous drainage parallels these arteries and drains in the portal system but is potentially more identifiable because the veins are about twice as large as the small epiploic arteries. These vessels may be clearly identified in 100 percent of patients [1].

Benign diseases of the GO essentially concern ischemic or mechanical entities. Segmental omental infarction/torsion of the GO is a rare but now well-known entity concerning the right portion of the GO in more than 90 percent of cases and simulates surgical emergencies such as cholecystitis, appendicitis, and diverticulitis. Left-side infarction is rare.

Torsion of the GO is a rare acute condition in which the organ twists around its long axis. GO torsion is classified as primary or secondary, the latter being more common and usually seen in the presence of a bulky abdominal tumor, hernia, or as sequelae of a surgical procedure such as Roux-en-Y gastric bypass. These pathological conditions may cause focal scarring and adhesions within the GO, predisposing the remainder of the mobile omentum to twist around this pivot. Internal hernias through a congenital or acquired defect of the GO have also been sporadically reported.

The reported case differs drastically from the situations remembered above. Here a large portion of the mesentery represented the pivot of an unusual encircling and incarcerating 360-degree complete whirl of the GO. It is likely that an adhesional field related to a previous surgery favored this omental twist.
